# Findings on Fluorine-18 Fluorodeoxyglucose Positron Emission Tomography/Computed Tomography in a Patient with Malignant Pheochromocytoma

**DOI:** 10.4274/MIRT.018253

**Published:** 2011-08-01

**Authors:** Pelin Ozcan Kara, Taylan Kara, Gonca Kara Gedik, Oktay Sari, Ozlem Sahin

**Affiliations:** 1 Selcuk University Selcuklu Medical Faculty, Department of Nuclear Medicine, Konya, Turkey; 2 Beyhekim State Hospital, Department of Radiology, Selcuklu, Konya, Turkey; 3 Selcuk University Meram Medical Faculty, Department of Nuclear Medicine, Konya, Turkey

**Keywords:** Pheochromocytoma; FDG PET/CT

## Abstract

Pheochromocytomas are rare tumors arising from chromaffin cells of the sympathoadrenal system and 85% of them are located in the adrenal medulla. Malignant pheochromocytomas account for 10% of all pheochromocytomas. Since clinical, biochemical and histopathological features can not reliably distinguish malignant from benign tumors, malignancy is established in the presence of distant metastases. Although in some cases, metastases may develop during follow-up, most of these tumors have metastatic disease at initial presentation. In this case report, detection of distant metastases and recurrence developed during follow-up with 18-flouro-deoxyglucose positron emission tomography/computed tomography (FDG PET/CT) in a patient with malignant pheochromocytoma was presented.

**Conflict of interest:**None declared.

## INTRODUCTION

Pheochromocytomas are rare tumors arising from chromaffin cells of the sympathoadrenal system and 85% of them are located in the adrenal medulla. Malignant pheochromocytomas account for 10% of all pheochromocytomas. Since clinical, biochemical and histopathological features can not reliably distinguish malignant from benign tumors, malignancy is established in the presence of distant metastases. In this case report, detection of distant metastases and recurrence developed during follow-up with 18-flouro-deoxyglucose positron emission tomography/computed tomography (FDG PET/CT) in a patient with malignant pheochromocytoma was presented.

## CASE REPORT

**Figure 1:**
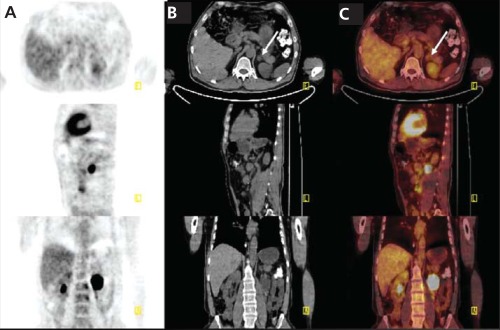
FDG PET/CT scan. Axial, sagittal and coronal PET (A), unenhancedCT (B), Fused (C) images illustrate a nodular lesion with mildlyincreased FDG uptake localized on left adrenal gland (arrows)(SUV_max_: 2.86)

**Figure 2:**
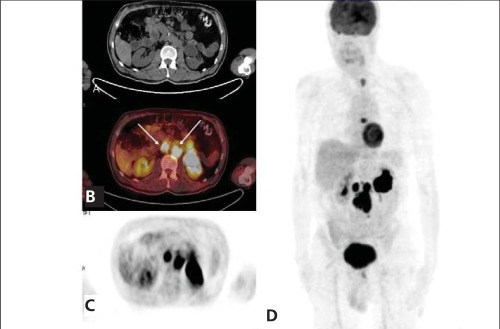
FDG PET/CT scan. Axial unenhanced CT (A), Fused (B), PET(C) and PET/CT MIP (D) images identified both recurrence at thesite of original disease and metastatic regions. Paraaortic andparacaval lymph nodes are shown with arrows

**Figure 3:**
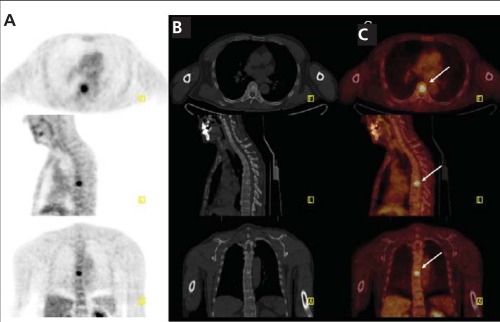
FDG PET/CT scan. Axial, sagittal and coronal PET (A), unenhancedCT (B), Fused (C) images illustrate metastatic sclerotic lesionon 7^th^ thoracal vertebra with increased FDG uptake (arrows)(SUV_max_: 9.23)

A 50 year old man with a history of pheochromocytoma for six years was presented with recurrence. He had biopsy proven pheochromocytoma and two operations for recurrence, the last of which was two years ago. Left adrenal soft tissue mass with a diameter of 11 mm and lymph nodes located at paraaortic-paracaval region were shown on CT imaging. Further functional imaging with FDG PET/CT depicted a nodular lesion with mildly increased FDG uptake localized on left adrenal gland which was not detected on his previous imaging exams (Figure 1 arrows). Maximum standardized uptake value (SUV_max_) of the lesion was calculated as 2.86. Increased FDG uptake (SUV_max_:19.44) in paraaortic and paracaval lymph nodes was also observed ( Figure 2 arrows). Additionally, metastatic sclerotic lesion on 7^th^ thoracal vertebra with increased FDG uptake (SUV_max_: 9,23 ) was detected (Figure 3 arrows).

## LITERATURE REVIEW AND DISCUSSION

Recently, FDG PET /CT have emerged as an important clinical diagnostic tool in a variety of malignancies. Whether benign or malignant, most pheochromocytomas are metabolically active and accumulate FDG. Shulkin et al. detected pheochromocytoma with FDG PET in 22 of 29 patients (76%) (1). Magnetic resonance imaging (MRI) provides excellent anatomic detail and is more accurate than CT especially in detecting extra-adrenal pheochromocytomas. MRI lacks in sensitivity as availability of whole-body imaging may be difficult. CT is accurate in detecting primary adrenal pheochromocytomas with high sensitivities, although specificity is low and less successful in detection of recurrent disease. Whole-body CT scanning is impractical for routine use due to radiation exposure. Therefore, CT is limited in the detection of multifocal disease. In a study by Timmers et al. FDG PET was found useful with a 100% per patient sensitivity and a 97% per body region sensitivity for pheochromocytomas, better than either CT or MRI and especially useful in the detection of distant metastasis (2). In a study by Mann et al, both C-11 HED and F-18 FDG were able to localize more lesions in a more timely fashion than I-131 MIBG (3). Several studies have shown that most pheochromocytomas accumulate FDG (1,2,3,4,5). However, FDG uptake is found in a greater percentage of malignant than benign pheochromocytomas (1). In this case, recurrent adrenal lesion, lymph node involvement and bone metastasis were successfully detected by FDG PET/CT. In cases whom extraadrenal and/or metastatic disease is suspected, and anatomical imaging results are equivocal or negative, FDG PET/CT should be used to assess the extent of disease.
